# Cognitive underpinnings of malevolent creativity: the role of problem construction

**DOI:** 10.3389/fpsyg.2026.1788182

**Published:** 2026-09-15

**Authors:** Averie Linnell, Roni Reiter-Palmon

**Affiliations:** ICON Lab, Psychology Department, University of Nebraska at Omaha, Omaha, NE, United States

**Keywords:** creative problem solving, malevolent creativity, problem construction, problem finding, workplace deviance

## Abstract

**Introduction:**

Malevolent creativity (MC), the generation of ideas that are both novel and intentionally harmful, poses significant risks in organizational settings due to its unexpected nature. While research has examined individual differences and situational factors that predict MC, cognitive processes underlying harmful ideation remain underexplored. Problem construction, the early-stage process of defining goals and parameters before generating solutions, is established as critical to creative performance, but its role in MC is unknown. This study examined whether problem construction fluency (the number of unique ways individuals reframe a problem) predicts the generation of harmful ideas, original ideas, and malevolently creative ideas (both harmful and original).

**Methods:**

Working adults (*N* = 155) recruited via Prolific responded to an ambiguous workplace vignette depicting unfair treatment. Participants first engaged in problem construction by generating multiple restatements of the problems, and then generated creative solutions. Solutions were rated for originality and harmfulness using the Consensual Assessment Technique by independent rater teams. Hierarchical regression analyses examined whether problem construction fluency predicted outcomes beyond the effects of control variables (age, impression management, problem-solving experience).

**Results:**

Problem construction fluency positively predicted original ideas (β = 0.41, *p* < 0.001), harmful ideas (β = 0.29, *p* < 0.001), and malevolent creativity (β = 0.26, *p* = 0.001). Impression management suppressed harmful ideation and MC but not originality. Older participants generated more original and malevolently creative ideas, but not more harmful ideas.

**Discussion:**

Findings suggest that MC may operate through similar cognitive processes as general creative problem solving rather than representing impulsive responses to provocation. Deeper engagement with the problem space during problem construction was associated with a higher likelihood of generating ideas that are original, harmful, or both. These results suggest MC may involve sophisticated cognitive processing that is associated with effortful engagement in early stages of the creative process.

## 1 Introduction

Two former employees of California's Caltrain system embezzled public funds to secretly convert train station office spaces into private apartments, complete with kitchens, showers, and security cameras (Johns, 2025). To avoid detection, they systematically suppressed invoice expenditures below the $3,000 threshold that would trigger managerial review, successfully implementing this strategy over several years. The district attorney emphasized the novelty of this crime, stating, “This is why I say to people, “never say you've seen it all,” because something new like this will come up” (Johns, 2025). The employees identified a problem (wanting free housing), explored the parameters and constraints of their environment (invoice thresholds, unused spaces), and generated an original solution that others would not anticipate.

This case illustrates how employees can harm organizations through deliberate engagement with harmful goals, such as “How can we embezzle public funding without getting caught?” Answering novel and complex questions is a hallmark of creative problem solving (Mumford et al., 1991). Creativity, or the ability to generate unique and effective ideas (Amabile, 1996; Mumford and Gustafson, 1988; Runco and Jaeger, 2012), provides valuable contributions to society, such as innovative products, efficiency gains, and breakthrough solutions to pressing societal problems. From a process view of creativity, three core phases consistently emerge: problem construction, idea generation, and idea evaluation and implementation (Lubart, 2001; Mumford et al., 1991). Through these phases, individuals define the context and parameters of a problem, generate ideas to address the problem, and evaluate those ideas so that the most effective one can be implemented. However, the same cognitive processes that produce valuable innovations can also be directed toward harmful ends instead. In the Caltrain embezzlement case, the financial harm totaled more than $40,000 of public funding.

Malevolent creativity (MC), defined as intentional harm achieved through novel and effective means (Harris et al., 2013), poses a significant risk to organizations. Any form of deviance with destructive goals can threaten an organization and its employees (Bennett and Robinson, 2003). However, the unexpected nature of MC makes it more difficult to detect and, as a result, potentially more harmful (Cropley et al., 2008; Harris et al., 2013). Despite growing recognition of MC's organizational relevance (d'Amato and Hunter, 2025), research has primarily focused on emotional and situational antecedents rather than cognitive processes underlying MC.

Of the limited research on cognitive processes in MC, researchers have prioritized the idea generation phase (e.g., Kapoor and Khan, 2017; Lee and Dow, 2011; Perchtold-Stefan et al., 2021). Understanding the full cognitive process is critical for predicting and preventing insider threats and sophisticated workplace deviance. In particular, the early stage process of problem construction, in which the problem solver establishes the context and goals of the problem-solving effort, warrants empirical investigation (Mumford et al., 1994; Reiter-Palmon et al., 1997).

The majority of prior research on MC has examined individual differences, such as trait aggression (Harris and Reiter-Palmon, 2015; Lee and Dow, 2011), and contextual factors, such as perceived injustice (Geng et al., 2024; James et al., 1999; Zhang et al., 2024). Hunter et al. (2022) proposed that implementing malevolent creative ideas requires both willingness (motivation) and ability (skills and resources) to explore harmful options. The extant literature on the cognitive underpinnings of MC suggests it shares mechanisms with benevolent or neutral creativity. MC correlates positively with general divergent thinking, or the ability to generate multiple, divergent ideas (Guilford, 1967; Hao et al., 2016; Perchtold-Stefan et al., 2021). Further, neuroimaging research reveals similar brain activation patterns for general and malevolent creative ideation (Perchtold-Stefan et al., 2023). However, research has focused almost exclusively on idea generation, leaving earlier cognitive processes unexplored.

This gap is notable given that MC theoretically operates through the same creative problem-solving process as benevolent creativity (Cropley et al., 2008; d'Amato and Hunter, 2025; Hunter et al., 2022). Moreover, the perpetrator's harmful intent is theorized to be present throughout each phase of the creative process (d'Amato and Hunter, 2025).

Problem construction is a core cognitive process in creative thought that defines the context for problem-solving by establishing goals, objectives, and parameters (Mumford et al., 1994; Reiter-Palmon et al., 1997). In real-world settings, problems are often complex and ill-defined, requiring individuals to first identify and construct the problem before generating solutions. This process involves perceiving environmental cues and drawing upon problem representations to frame the current situation. These problem representations are knowledge structures based on past experiences (Holyoak, 1984).

When facing ambiguous workplace situations, cues may trigger multiple problem representations simultaneously. The more complex the situation, the more competing representations must be considered and integrated (Mumford et al., 1994). These competing demands and tensions require effortful cognitive processing through problem construction to generate creative solutions (Miron-Spektor et al., 2018; Steele et al., 2021). Importantly, considering and integrating more competing representations requires greater cognitive effort to resolve tensions between different ways of framing the problem and is associated with more original solutions (Reiter-Palmon et al., 2023).

Problem construction fundamentally shapes the creative process because the framing of a problem determines what solutions are considered viable (Mumford et al., 1994). By reframing a problem in multiple ways, individuals explore different goal structures and consider diverse approaches to resolution. This ability to see a problem from multiple perspectives is associated with generating more creative solutions because it expands the solution space beyond initial, obvious responses (Reiter-Palmon et al., 2023; Reiter-Palmon and Robinson, 2009).

While problem construction has been established as critical to creative problem-solving broadly (Mumford et al., 1994; Reiter-Palmon et al., 2023), its role in MC remains largely unexplored. The absence of empirical investigation in this area represents a significant gap, and recent theoretical work explicitly calls for research on problem construction in the MC process (Perchtold-Stefan et al., 2025). Theoretically, the relationship between problem construction and malevolent creativity could operate in opposing directions.

One argument is that extensive problem construction may facilitate MC through expanded exploration of the problem space. When facing an unfair workplace situation, an individual might construct the problem as “How can I improve my working conditions?” (benevolent), “How can I document this injustice?” (neutral), or “How can I retaliate without detection?” (malevolent). Engaging further in problem construction leaves more room for diverse problem framing and may be associated with a higher likelihood that a problem solver will explore harmful goal structures. Moreover, MC requires the cognitive sophistication to identify unconventional approaches toward the goal of harm. Simple, obvious, harmful acts (e.g., yelling at a colleague, damaging property) are easily detected and deterred through organizational policies and social norms. In contrast, truly creative harm, and creativity in general, requires deeper engagement with the problem space to identify novel goals and opportunities (d'Amato and Hunter, 2025; Mumford et al., 1994). This engagement with constraints and goals would primarily occur during the problem-construction phase (Mumford et al., 1991; Reiter-Palmon et al., 1997).

Conversely, deliberate cognitive engagement through problem construction may function as constructive coping (Carson and Runco, 1999; Leone, 2024), such that frustration may be expressed through effective problem-solving rather than impulsive retaliation. Carson and Runco (1999) found that problem generation ability was negatively related to confrontive and escape-avoidance coping strategies. The authors suggest that individuals who engage actively in problem finding are able to respond to stressful situations in more adaptive ways. This finding is in line with research that posits creativity as a high-level coping strategy that can allow for successful adaptation (Smith and Van Der Meer, 1990). Problem construction also shares similarities with cognitive reappraisal from the emotion regulation literature. Cognitive reappraisal is the process of reinterpreting an emotional or stressful situation from multiple perspectives and reframing situations more positively (Gross and John, 2003). Cognitive reappraisal can effectively regulate anger in response to anger-provoking events (Mauss et al., 2007), like the injustice provocations described in our vignettes.

Although problem construction occurs automatically, deliberate, effortful processing can move individuals beyond their initial reactions, drawing on past experiences to consider more complex or future-oriented approaches (Mumford et al., 1996; Reiter-Palmon et al., 1998). Deeper engagement may be associated with individuals reframing situations in ways that identify appropriate solutions rather than harmful ones. From this perspective, individuals who engage more extensively in problem construction may generate less harmful responses. Through more thorough exploration of the problem space, individuals may discover constructive alternatives they would have otherwise overlooked or recognize that harmful approaches carry risks and consequences that outweigh their appeal. This view aligns with early theoretical work suggesting that MC may primarily reflect impulsive, emotional responses to provocation (Harris and Reiter-Palmon, 2015; James et al., 1999). If deliberate cognitive engagement through problem construction is associated with more considered responses, harmful impulses may be less likely to manifest, with more constructive outcomes observed instead.

It is important to note that what constitutes an “appropriate” response may itself be shaped by individual differences (e.g., aggression), with some individuals viewing retaliation as a legitimate problem-solving strategy (Harris, 2013; Harris et al., 2014). This consideration highlights why examining engagement in problem construction is particularly relevant to understanding MC. Problem construction is the phase during which the problem solver establishes goals and forms intentions (Mumford et al., 1994; Reiter-Palmon and Robinson, 2009). If malevolent creativity is defined in part by harmful intent (d'Amato and Hunter, 2025; Harris et al., 2013), then the intention to harm is embedded in the problem-construction phase. Individuals who frame a provocative situation as “How can I get revenge?” have already established a harmful goal structure during problem construction.

These competing perspectives have different implications for understanding MC. If problem construction facilitates MC, this would support the view that MC arises from the same neutral cognitive processes that underlie creative problem solving more broadly, with differences driven by the goals pursued or constraints emphasized rather than by distinct cognitive operations. If problem construction reduces MC, this would suggest that engagement in the cognitive phases of problem-solving differs fundamentally between instances in which the goal is benevolent vs. harmful. The present study is one of the first to examine these possibilities empirically.

To test whether problem construction predicts MC, we developed workplace-specific vignettes depicting ambiguous, unfair situations. Previous MC research has primarily used explicit “revenge” tasks that instruct participants to “get back at” someone (Harris and Reiter-Palmon, 2015; Perchtold-Stefan et al., 2021) or generate uses for objects that could harm others. While useful for eliciting MC responses, these approaches conflate task compliance with genuine malevolent ideation and specify an explicit harm target in advance. Our approach differs in three important ways. First, we used realistic workplace scenarios rather than abstract tasks, improving ecological validity and respondent motivation (Hughes et al., 2018; Plucker, 2017). Second, our vignettes did not explicitly instruct participants to generate harmful ideas, allowing us to observe whether problem construction naturally leads to malevolent ideation in workplace contexts. Third, our vignettes did not specify a target of harm. This design choice was to allow for a complex and ambiguous situation where participants are required to engage in problem construction to determine the goals, constraints, and parameters on their own. Excluding a predefined target allows participants to decide if they will pursue a constructive or destructive solution, which more closely resembles real-world workplace situations.

We developed workplace-specific vignettes depicting unfair situations to reflect perceived injustice, a theoretical and empirical antecedent of MC in the workplace (Geng et al., 2024; James et al., 1999; Zhang et al., 2024). Two vignettes were developed for use in the study. One vignette described a data scientist experiencing unfair treatment from their manager compared to their coworkers, miscommunication with other teams, and redoing work tasks. The second vignette described a nurse who has been dealing with understaffing, budget cuts, disrespectful doctors, and a lack of employee appreciation from management. An important tradeoff of this approach, however, is that the absence of an explicit harm target increases the difficulty of reliably classifying harmful intent in generated solutions and reduces the base rate of MC ideas. We discuss these limitations in the Discussion section.

We asked participants to engage in problem construction by reframing workplace problems in multiple ways before generating solutions. We then examined whether the number of unique problem restatements generated (i.e., problem construction fluency) predicted three outcomes: harmful ideas, original ideas, and malevolent creativity (ideas that are both original and harmful).

Based on established findings linking problem construction to creative performance (Mumford et al., 1994; Reiter-Palmon and Robinson, 2009), we hypothesized:

Hypothesis 1: Problem construction fluency will positively predict the generation of original ideas, such that a greater number of problem restatements will be associated with a greater number of original solutions.

For harmful ideas and malevolent creativity, competing theoretical perspectives preclude directional predictions. Extensive problem construction could increase exploration of the problem space, potentially including harmful goals. When facing an unfair workplace situation, an individual who generates more problem frames may be more likely to encounter malevolent framings (e.g., “How can I retaliate without detection?”) alongside constructive ones. Because malevolent creativity is theorized to share cognitive mechanisms with benevolent creativity (Cropley et al., 2013; d'Amato and Hunter, 2025), the same processes that enhance originality might also facilitate harmful ideation.

On the other hand, deliberate cognitive engagement through problem construction may function as constructive coping, such that individuals who engage more extensively in problem construction may be more likely to express frustration through effective problem-solving rather than impulsive retaliation. From this perspective, individuals who engage more deeply in problem construction may generate less harmful responses by reframing situations to identify appropriate solutions. We therefore posed the following exploratory research questions:

Research Question 1: What is the relationship between problem construction fluency and the generation of harmful ideas?
Research Question 2: What is the relationship between problem construction fluency and malevolent creativity (ideas that are both original and harmful)?

Additionally, we controlled for problem-solving experience, age, and impression management (social desirability), as these variables may influence both problem construction engagement and willingness to report harmful ideas. We include more information on the selected covariates in the measures section.

## 2 Methods

### 2.1 Participants

We conducted an *a priori* power analysis using G^*^Power 3.1 (Faul et al., 2007) to determine the minimum sample size needed to detect effects in hierarchical multiple regression. Based on previous research examining problem construction and creativity (Reiter-Palmon et al., 1997), we anticipated a medium effect size (*f*^2^ = 0.15, corresponding to *R*^2^ ≈ 0.13). To achieve 80% power to detect this effect with α = 0.05 and up to four predictors in the final model, we required a minimum sample size of 85 participants.

We recruited working adults aged 19 years or older living in the United States via Prolific, an online research platform. Participants were compensated $2.00 for their time (approximately $8.00/hour) if they chose to participate. A total of 361 participants completed the study. Ten participants were removed from analyses for failing to meet data quality standards. Four were full-time students, three did not meet employment eligibility criteria, and three were removed for low-quality responses or implausibly short completion times. 170 participants were randomly assigned to the data scientist task and 181 participants were randomly assigned to the nurse task. The final analytic sample for the data scientist task is *n* = 155, which reflects listwise deletion due to missing data on the AUT (*n* = 3) problem construction responses (*n* = 9) and idea generation tasks (*n* = 2), or both (*n* = 1). For the nurse vignette, participants were excluded for missing AUT responses (*n* = 3), idea generation task (*n* = 8) and the problem construction task (*n* = 20) leaving a final sample of *n* = 150. Participants with missing problem-construction data provided responses that the raters were unable to code (did not follow directions). All other study variables (impression management, age, demographics) had complete data. The final sample included 305 participants across two vignette conditions with 155 assigned to the data scientist vignette and 150 to the nurse vignette.

As described in the Introduction, both vignettes were designed to be ambiguous and complex with no clearly defined target. However, the nurse vignette proved to be overly complex, which caused issues for assessing the valence of responses. Because the nurse vignette described a multi-stakeholder systemic problem, virtually all solutions carried unintended consequences for competing stakeholders. For example, raters were divided on whether responses such as walkouts, unionizing, reporting to authorities or news outlets, or getting employees fired should be considered harmful or beneficial. A walkout could be interpreted as forcing administration to address dangerous working conditions (beneficial) or as further jeopardizing patient care (harmful). These inherent ambiguities reflect a genuine feature of complex systemic problems: most solutions both help and harm depending on the perspective taken. Although realistic, this vignette was not conducive to reliably observing harmful intent in the present study design.

Although inter-rater reliability was initially acceptable for the nurse vignette following the 10% training set, the full ratings did not meet our quality threshold [ICC (2,3) ≥ 0.70], and additional rater training was required. Even after retraining, the final valence ratings showed an extremely leptokurtic distribution clustering at the neutral midpoint.

Following the modified CAT (Amabile, 1982; Reiter-Palmon, 2021), raters are instructed to rate only on what participants wrote and not to “read into” responses. When harm and benefit are genuinely indeterminate across stakeholder perspectives, neutral ratings are the defensible default under CAT instructions that discourage raters from inferring intent beyond what is explicitly stated. We discuss the issue of inferring intent in MC research in the Discussion.

The vignette's complexity effectively created a ceiling on the discriminability of harmful intent, limiting variance in valence ratings. This range restriction was not conducive to count-based MC threshold classifications. Notably, the mean valence ratings for the nurse vignette were higher than in the data scientist vignette. However, this elevated mean was a consequence of the leptokurtic distribution. When ratings cluster heavily at the neutral midpoint with a small number of extreme responses, the resulting mean overestimates the central tendency of the distribution and is not a valid basis for threshold-based MC scoring. We therefore excluded the nurse vignette from primary analyses. The decision to exclude the nurse vignette was based on concerns of the vignette's discriminatory validity rather than an absence of harmful ideation. We address important design considerations for future MC research based on this finding in the Discussion section. Exploratory analyses using the nurse vignette are reported in the supplemental analyses section.

The current analyses focus on the participants randomly assigned to the data scientist vignette. Our final sample of 155 participants provided high power (0.98) to detect medium effects (*f*^2^ = 0.15) and sufficient power (0.89) to detect smaller effects (*f*^2^ = 0.10, corresponding to *R*^2^ ≈ 0.09). *Post-hoc* sensitivity analyses confirmed that with *N* = 155, α =0.05, and 80% power, we could reliably detect effect sizes as small as *f*^2^ = 0.08 (*R*^2^ ≈ 0.07) in models with up to four predictors.

Participants' ages ranged from 20 to 85 years (*M* = 40.21, *SD* = 12.67). The sample was approximately evenly split by gender (49.0% women, 47.7% men, 2.6% non-binary) and predominantly White (72.3%), with Black or African American, Asian, and multiracial participants each comprising approximately 7–9% of the sample. Participants represented a variety of industries, with the most common being office jobs (17.4%), healthcare (12.3%), and management or human resources (10.3%). Full demographic information is presented in Table 1.

**Table 1:** Demographic characteristics of participants.

Characteristic	*n*	Percent
Gender		
Man	74	47.7
Woman	76	49.0
Non-binary	4	2.6
Prefer not to say	1	0.6
Race/Ethnicity		
Asian	10	6.5
Black/African American	14	9.0
Hispanic or Latino	4	2.6
Hawaiian/Pacific Islander	0	0.0
Middle Eastern or North African	1	0.6
Native American or Alaska Native	0	0.0
White	112	72.3
Multiracial/multiethnic	12	7.7
Other	2	1.3
Education level		
Less than high school	2	1.3
High school degree or equivalent	15	9.7
Some college but no degree	25	16.1
Associate's degree	21	13.5
Bachelor's degree	56	36.1
Master's degree	28	18.1
Doctoral or professional degree	8	5.2
Employment status		
Employed full-time	125	80.6
Employed part-time	20	12.9
Self-employed	10	6.5
Industry		
Retail	12	7.7
Food services	9	5.8
Accounting/finance	8	5.2
Management/HR	16	10.3
Office	27	17.4
Healthcare	19	12.3
Sports/fitness	1	0.6
Academic	14	9.0
Marketing/sales	10	6.5
Service	9	5.8
Other	30	19.4

*N* = 155.

### 2.2 Measures for assessing data quality

Five instructional attention checks were randomly presented throughout the survey to assess careless responses. Further, open-ended responses were assessed for nonsensical answers, copying and pasting information from online, or failing to follow directions. We also assessed response time and removed responses with impossibly low or excessively long completion times from analyses.

We explicitly prompted participants not to use AI chatbots for the open-ended vignettes tasks. To further dissuade participants from using AI, the copy-and-paste functions in the Qualtrics survey were disabled. That is, participants were unable to copy the prompt from the survey or paste AI-generated responses back into it. Of course, participants could still type everything by hand instead of copying and pasting, but this extra effort was intended to discourage copying responses.

### 2.3 Procedure

We informed participants that the purpose of the study was to investigate problem-solving at work, as well as their attitudes and behaviors. We obtained consent by presenting participants with a cover letter, informing them that their participation is voluntary, and asking them to consent by proceeding. After consenting, participants were presented with the ambiguous workplace vignette and asked to generate as many creative solutions as they could. We did not explicitly instruct participants to engage in malevolent behaviors. Following the idea generation task, participants completed a measure of social desirability. Lastly, participants completed a demographics questionnaire.

### 2.4 Measures

#### 2.4.1 Problem construction task

We presented participants with the ambiguous workplace vignette and asked them to imagine themselves in the scenario. In order to elicit engagement in problem construction, we instructed participants to “restate the problems in the scenario in your own words as many different ways as you can, starting each statement with ‘How can I' or ‘How should I'.” We scored responses to the problem-construction task for fluency, i.e., the number of unique restatements generated by each participant.

#### 2.4.2 Malevolent creativity

We asked participants to generate as many creative solutions as they could in response to the ambiguous problem. Independent teams of three raters rated the originality and valence of the generated solutions following a modified version of the Consensual Assessment Technique (CAT; Amabile, 1982, 1996). The raters were trained undergraduate and graduate research assistants familiar with the CAT. The first author met with each rater team to direct additional training specific to the dataset and rating scales. Following training, raters independently rated 10% of the full dataset. After checking intraclass correlations (ICCs; Shrout and Fleiss, 1979) for acceptable reliability, the raters moved on to the full dataset.

##### 2.4.2.1 Originality

Originality is defined as the relative novelty of an idea. Originality was rated from 1 (*very unoriginal*) to 5 (*very original*). For the full scale, see Appendix A. The ICC (2, k) indicated acceptable agreement [ICC (2, 3) = 0.79]. We averaged the scores across the three raters to create a single originality score for each solution. Ideas that scored an average score above 3.00 were considered original because 3 on the originality scale represents a neutral mid-point. We calculated the total number of original ideas per person.

##### 2.4.2.2 Valence

Raters assessed the degree of benevolence or malevolence of solutions using a valence scale. Malevolent solutions intentionally cause physically or psychologically harmful outcomes, and benevolent solutions intentionally cause physically or psychologically helpful outcomes. The scale anchors ranged from 1 (*very benevolent*) to 5 (*very malevolent*), with a neutral point at 3. For the full scale, see Appendix B. The ICC (2, k) indicated acceptable agreement [ICC (2, 3) = 0.76]. Again, we averaged the scores across the three raters to create a single valence score for each solution. We considered a solution harmful if it averaged above 3.00 on valence. This cutoff is consistent with the scale's explicit neutral midpoint and scoring convention established in the MC literature (Harris and Reiter-Palmon, 2015; Kapoor and Khan, 2017). We calculated the total number of harmful ideas for each individual, following established operationalizations (Harris and Reiter-Palmon, 2015).

#### 2.4.3 Demographics

The demographics questionnaire included age, gender, and racial and ethnic identity. Further, we surveyed employment status, level of education, job industry, and tenure.

#### 2.4.4 Covariates

We identified problem-solving experience, age, and impression management as potential covariates based on their theoretical relevance to creative ideation and harm-related responses. Because prior experience can shape how individuals interpret and approach novel problems (Holyoak, 1984; Mumford et al., 1994; Reiter-Palmon et al., 1998), we developed a brief measure to assess familiarity with the vignette problems and to control for prior exposure to similar problem-solving contexts. We included problem-solving experience as a covariate for all models. We included age in our models for originality and malevolent creativity because older individuals have more extensive problem-solving experience, which forms the foundation for problem representations activated during problem construction (Holyoak, 1984; Mumford et al., 1994). This accumulated experience may support more sophisticated problem framing and more diverse solution generation, consistent with previous research on creativity and aging, which suggests accumulated knowledge and life experience can support tasks that require deep, structural knowledge (Heilman and Fischler, 2018). Problem construction in complex, ambiguous workplace scenarios may benefit from crystallized intelligence because it requires drawing on accumulated knowledge relevant to the problem scenario. Finally, social desirability is particularly relevant when studying harmful ideation, as individuals may withhold malevolent ideas because they violate social norms (Hao et al., 2016; Kapoor and Khan, 2017; Reiter-Palmon, 2018). We included impression management, the tendency to deliberately present oneself favorably to others (Paulhus and Reid, 1991), in our model predicting harmful ideas and malevolently creative ideas to address this concern.

We assessed social desirability using the Balanced Inventory of Desirable Responding (BIDR; Paulhus and Reid, 1991) short-form version (BIDR-6; Asgeirsdottir et al., 2016). The Impression Management subscale consists of 10 items assessing the tendency to deliberately present oneself in a socially desirable manner by tailoring responses to perceived social norms (α = 0.71). A sample item is “I sometimes tell lies if I have to” (reverse-scored). Participants rated items on a 7-point Likert-type scale ranging from 1 (*not true*) to 7 (*very true*). Negatively keyed items were recoded prior to computing scores, with higher scores indicating a greater propensity to engage in impression management. The Problem-Solving Experiences Questionnaire consists of five items (α = 0.85) that reflect past experiences related to the present problem-solving task, rated on a 5-point Likert-type scale from 1 (*strongly disagree*) to 5 (*strongly agree*). A sample item is “I have solved this kind of problem.”

## 3 Results

On average, participants generated 3.26 problem restatements (*SD* = 1.44), with 0.38 harmful ideas (*SD* = 0.76), 0.35 original ideas (*SD* = 0.83), and 0.14 malevolently creative ideas that were both original and harmful (*SD* = 0.52). Problem construction fluency was positively correlated with harmful ideas (*r* = 0.30, *p* < 0.001), original ideas (*r* = 0.40, *p* < 0.001), and malevolent creativity (*r* = 0.26, *p* = 0.001). Descriptive statistics and correlations among study variables are presented in Table 2.

**Table 2:** Descriptive statistics and correlations among study variables.

Variable	M	SD	1	2	3	4	5	6
1. Harmful ideas	0.38	0.76	—					
2. Original ideas	0.35	0.83	0.48^***^	—				
3. Malevolent creativity	0.14	0.52	0.75^***^	0.70^***^	—			
4. PC fluency	3.26	1.44	0.30^***^	0.40^***^	0.26^**^	—		
5. Impression management	4.49	0.70	−0.18^*^	−0.10	−0.17^*^	−0.06	—	
6. Age	40.21	12.67	0.14	0.26^***^	0.22^**^	−0.04	0.07	—
7. DT fluency	4.92	2.50	0.24^**^	0.27^***^	0.19^*^	0.46^***^	0.01	0.17^*^

*N* = 155. PC, problem construction; DT, divergent thinking. ^*^p < 0.05. ^**^p < 0.01. ^***^p < 0.001.

### 3.1 Model 1 results: harmful idea generation

To examine predictors of harmful idea generation, we conducted a hierarchical regression analysis. In Step 1, impression management and problem-solving experience were entered as control variables and accounted for 4.7% of the variance in harmful ideas, *F*_(2, 152)_ = 3.76, *p* = 0.026. In Step 2, problem construction fluency was added, which significantly improved model fit, Δ*R*^2^ = 0.08, Δ*F*_(1, 151)_ = 14.42, *p* < 0.001. The final model was significant, *F*_(3, 151)_ = 7.53, *p* < 0.001, *R*^2^ = 0.13 (see Table 3).

**Table 3:** Hierarchical regression analyses predicting harmful, original, and malevolently creative ideas.

	Model 1: harmful ideas		Model 2: original ideas		Model 3: malevolently creative ideas	
Predictor	β	*p*	β	*p*	β	*p*
Step 1						
Impression management	−0.19	0.021	—	—	−0.18	0.021
Age	—	—	0.26	0.001	0.23	0.003
PSE	−0.12	0.143	0.02	0.824	−0.04	0.625
*R^2^*	0.05		0.07		0.08	
*F*	3.76	0.026	5.64	0.004	4.49	0.005
Step 2						
Impression management	−0.17	0.030	—	—	−0.17	0.029
Age	—	—	0.28	<0.001	0.24	0.002
PSE	−0.12	0.103	0.00	0.952	−0.05	0.543
PC fluency	0.29	<0.001	0.41	<0.001	0.26	0.001
*R^2^*	0.13		0.24		0.15	
*F*	7.53	<0.001	15.52	<0.001	6.43	<0.001
*ΔR^2^*	0.08		0.17		0.06	
*ΔF*	14.42	<0.001	32.90	<0.001	11.32	0.001

*N* = 155. PC, problem construction; PSE, problem solving experience.

Problem construction fluency emerged as the strongest predictor of harmful ideas (β = 0.29, *p* < 0.001), indicating that participants who generated more problem restatements also produced more harmful solutions. Impression management was negatively associated with harmful ideas (β = −0.17, *p* = 0.030), suggesting that individuals higher in impression management reported fewer harmful responses.

### 3.2 Model 2 results: original idea generation

To examine predictors of original idea generation, we conducted a hierarchical regression analysis. In Step 1, we entered age and problem-solving experience as control variables, which accounted for 6.9% of the variance, *F*_(2, 152)_ = 5.64, *p* = 0.004. In Step 2, we added problem construction fluency, which significantly improved the model, Δ*R*^2^ = 0.17, Δ*F*_(1, 151)_ = 32.90, *p* < 0.001. The final model was significant, *F*_(3, 151)_ = 15.52, *p* < 0.001, *R*^2^ = 0.24 (see Table 3).

Problem construction fluency was the strongest predictor of original ideas (β = 0.41, *p* < 0.001), such that generating multiple problem restatements was associated with producing more original solutions. Age also significantly predicted originality (β = 0.28, *p* < 0.001), with older participants generating more original ideas.

### 3.3 Model 3 results: malevolently creative idea generation

To examine predictors of malevolent creativity, we conducted a hierarchical regression analysis. In Step 1, we entered impression management, age, and problem-solving experience as control variables, which accounted for 8.2% of the variance, *F*_(3, 151)_ = 4.49, *p* = 0.005. In Step 2, we added problem construction fluency, which significantly improved the model, Δ*R*^2^ = 0.06, Δ*F*_(1, 150)_ = 11.32, *p* = 0.001. The final model was significant, *F*_(4, 150)_ = 6.43, *p* < 0.001, *R*^2^ = 0.15 (see Table 3).

Problem construction fluency significantly predicted malevolent creativity (β = 0.26, *p* = 0.001), indicating that reframing the problem in multiple ways was associated with generating more ideas that were both original and harmful. Among control variables, age showed a positive relationship with malevolent creativity (β = 0.24, *p* = 0.002), with older participants generating more malevolently creative ideas. Impression management was negatively associated with malevolent creativity (β = −0.17, *p* = 0.029), suggesting social desirability suppressed the generation of malevolently creative solutions.

### 3.4 Supplemental analyses

Because we measured both problem construction engagement and MC ideation using fluency metrics, general ideational fluency could be a confound. To address this concern, we conducted a supplemental hierarchical regression controlling for divergent thinking (DT) fluency measured via an Alternative Uses Task (AUT; pants or shoe object, randomly assigned). DT fluency scores were combined across the two AUT versions, which had comparable means (version 1: *M* = 4.88; version 2: *M* = 4.97). In Step 1, impression management, age, and DT fluency were entered as covariates. In Step 2, PC fluency was added. PC fluency remained a significant predictor of MC above and beyond DT fluency (β = 0.23, *p* = 0.008), while DT fluency was not significant (β = 0.05, *p* = 0.589). The unique contribution of PC fluency accounted for 4.1% of the variance in MC (*f*^2^ = 0.05). These results suggest that the effect of PC fluency on MC is not attributable to general ideational fluency. However, we note that the observed power for the PC fluency unique effect was 0.77, slightly below the conventional 0.80 threshold.

We also conducted exploratory analyses using the nurse vignette (*N* = 150). We decided to report the results for transparency, but they should be interpreted cautiously, given the scoring challenges described in the Method. We followed the same hierarchical regression structure as the analyses for the data scientist vignette.

For harmful ideas, impression management and problem-solving experience were entered in Step 1 and did not account for significant variance, *R*^2^ = 0.00, *F*_(2, 147)_ = 0.34, *p* = 0.715. PC fluency was added in Step 2 and did not significantly improve model fit, Δ*R*^2^ = 0.02, Δ*F*_(1, 146)_ = 2.87, *p* = 0.093. PC fluency was not a significant predictor of harmful ideas (β = 0.14, *p* = 0.093).

For original ideas, age and problem-solving experience were entered in Step 1 and did not account for significant variance, *R*^2^ = 0.02, *F*_(2, 147)_ = 1.39, *p* = 0.253. Adding PC fluency in Step 2 significantly improved model fit, Δ*R*^2^ = 0.03, Δ*F*_(1, 146)_ = 4.64, *p* = 0.033. PC fluency was a significant predictor of original ideas (β = 0.14, *p* = 0.033), but the overall model was not significant, *F*_(3, 146)_ = 2.49, *p* = 0.062.

For MC, impression management, age, and problem-solving experience were entered in Step 1 and did not account for significant variance, *R*^2^ = 0.02, *F*_(3, 146)_ = 1.07, *p* = 0.364. Adding PC fluency in Step 2 did not significantly improve model fit, Δ*R*^2^ = 0.01, Δ*F*_(1, 145)_ = 2.11, *p* = 0.148. PC fluency was not a significant predictor of MC (β = 0.12, *p* = 0.148).

We also considered multiple analytic strategies to account for the low base-rate of count-based outcomes and the positive skew of MC. Only 13 participants (8.4%) generated at least one malevolently creative idea (*M* = 0.14, *SD* = 0.52). Poisson regression is appropriate for discrete count dependent variables (Coxe et al., 2009). However, our data violated the equidispersion assumption of Poisson regression with a variance approximately twice the mean (*s*^2^ = 0.27). When the variance is larger than the mean, Negative Binomial regression is recommended. This model failed to converge because a dispersion parameter cannot be estimated with so many individuals generating zero malevolently creative ideas. A zero-inflated model is recommended if there is an overwhelming number of zeros, but with only 13 non-zero cases, the model was not estimable. We therefore retained our OLS regression analyses, which are robust to non-normality.

We identified heteroscedasticity in all three models using Breusch-Pagan tests (*p* = 0.002, <0.001, and 0.011; Koenker, 1981). As a robustness check, we re-estimated each model using heteroscedasticity-consistent (HC3) standard errors (Hayes and Cai, 2007). PC fluency remained significant in every model (*p* = 0.003, <0.001, and.022). We also examined high-leverage cases using the conventional threshold of 3(k + 1)/n. Three high-leverage cases per model were identified, however, excluding them did not substantially change our results (β = 0.25, 0.34, and.17; all *p* < 0.05). No observation approached the conventional influence threshold of Cook's *D* > 1 (maximum *D* = 0.28, 0.72, and 0.33).

## 4 Discussion

As hypothesized, problem construction fluency positively predicted the generation of original ideas (H1), replicating established findings linking problem construction to creative performance (Mumford et al., 1994; Reiter-Palmon et al., 1997). Regarding our research questions, we found that problem construction fluency also positively predicted both harmful ideas (RQ1) and malevolent creativity (RQ2). These findings suggest that the cognitive processes supporting creative problem solving are associated with harmful ideation as well. Deeper engagement with the problem space was associated with a higher likelihood of generating ideas that are original, harmful, or both.

Across all three models, problem construction fluency emerged as a consistent and significant predictor, with the strongest effects for original ideas (β = 0.41, *p* < 0.001), followed by harmful ideas (β = 0.29, *p* < 0.001) and malevolent creativity (β = 0.26, *p* = 0.001). In each case, adding problem construction fluency significantly improved model fit beyond control variables alone (Δ*R*^2^ = 0.06–0.17, all *p*s ≤ 0.001). Age predicted original ideas and malevolent creativity, with older participants generating more original and malevolently creative ideas. Impression management suppressed harmful ideation and malevolent creativity but did not significantly relate to originality alone, suggesting that social desirability primarily affects the willingness to report harmful content.

The exploratory analyses using the nurse vignette produced a weaker and less consistent pattern of results compared to the primary data scientist vignette analyses. PC fluency significantly predicted original ideas (β = 0.17, *p* = 0.033) but was not a significant predictor of harmful ideas (*p* = 0.093) or malevolent creativity (*p* = 0.148). These results could reflect a genuine boundary condition, meaning the relationship between PC fluency and MC may not generalize to all workplace contexts. Conversely, the different findings may be a measurement artifact reflecting the documented challenges in scoring valence for the nurse vignette. Given the issues with the nurse vignette, these findings should be interpreted cautiously.

To address the concern that PC fluency may primarily capture general ideational fluency rather than problem construction engagement specifically, we conducted supplemental analysis controlling for divergent thinking fluency measured via an independent Alternative Uses Task. PC fluency remained a significant predictor of MC above and beyond divergent thinking fluency (β = 0.23, *p* = 0.008), while divergent thinking fluency was non-significant. These results suggest that the effect is not solely attributable to general verbal production.

These findings have important theoretical implications for understanding the nature of MC. The positive relationship between problem construction and MC observed in the primary analyses is consistent with the view that malevolent creativity may draw on similar cognitive processes to benevolent creativity (Cropley et al., 2008; Hunter et al., 2022), rather than representing a fundamentally distinct process. If MC were primarily an impulsive, emotionally driven response to provocation, we would not expect systematic engagement in problem construction to be associated with harmful ideation. Instead, our findings suggest that MC may be associated with the kind of deliberate cognitive elaboration that characterizes creative problem solving more broadly.

This perspective is consistent with recent theoretical work positioning MC within the broader creative problem-solving process (d'Amato and Hunter, 2025; Perchtold-Stefan et al., 2025). The intention to harm, which is definitional to MC (Harris et al., 2013), may be established during problem construction when individuals frame situations around harmful goals. The primary findings contrast with early theoretical work suggesting that malevolent creativity may primarily reflect impulsive, emotional responses to provocation (Clark and James, 1999; James et al., 1999). While unfair and provocative situational factors may indeed motivate intentions to cause harm, the present findings suggest that deliberate cognitive engagement through problem construction may be implicated in true MC ideation in the context of interpersonal workplace provocation.

### 4.1 Limitations and future directions

An important limitation of the present study is its correlational design, which precludes causal conclusions about the relationship between problem construction and MC. While our findings are consistent with the interpretation that deeper engagement in problem construction is associated with the generation of harmful and original ideas, future research should rule out alternative explanations. For instance, individuals who have already formed harmful intentions may naturally generate more problem restatements oriented toward achieving those goals. From this perspective, the observed relationship could reflect goal-directed elaboration rather than problem construction, leading to the discovery of harmful framings. It may be that individuals motivated to cause harm are willing to put more deliberate effort into the problem-construction phase.

Future research should therefore employ experimental designs to clarify the directionality of the relationship between problem construction and MC. For instance, researchers could manipulate goal framing by assigning participants to harm-oriented versus neutral or prosocial goals prior to engaging in problem construction, and then examine how these goals shape subsequent restatements and ideation. Alternatively, researchers could experimentally manipulate problem construction itself by varying instructions. Participants could be randomly assigned to either proceed directly to idea generation without explicit problem construction or be instructed to generate multiple problem restatements before ideation. Investigating whether problem construction causally facilitates MC or whether harmful intentions drive deeper problem construction is an important next step.

It is important to acknowledge the low base rate of malevolently creative responses in our sample. On average, participants generated only 0.14 malevolently creative ideas (*SD* = 0.52), with the majority of participants producing zero ideas that met criteria for both originality and harmfulness. This floor effect is consistent with prior MC research, which has noted that ideas qualifying as both novel and harmful are rare, particularly when harmful ideation is not explicitly prompted (Harris and Reiter-Palmon, 2015; Kapoor and Khan, 2017). There are two related considerations that may have led to the distributional constraints. First, social desirability may have led participants to withhold harmful ideas even if they did generate them (which led to the inclusion of impression management as a covariate). We did find that impression management was negatively associated with harmful ideation and malevolent creativity within our models. Second, our vignette did not explicitly instruct participants to generate harmful ideas. Although this decision improves ecological validity, it is known to reduce the frequency of malevolent responses compared to explicit revenge task designs (Harris and Reiter-Palmon, 2015).

While this distribution may constrain the magnitude of detectable effects and limit variability in the outcome, the significant relationships we observed despite this constraint suggest that problem-construction fluency is a meaningful predictor of MC. The skewed distribution and limited variance in MC likely attenuated the magnitude of the observed relationships, making it more difficult to detect effects and constraining the range of explainable variance in regression models. Because of this limitation, we echo the call for a task designed specifically to observe problem construction in MC to elicit sufficient MC responses to draw more conclusions (Perchtold-Stefan et al., 2025).

Another limitation concerns our operationalization of MC. It was necessary to dichotomize the continuous originality and harmfulness variables to compute a final MC score because each scale had a neutral midpoint. Ideas that score above the midpoint on both dimensions are considered meaningfully original and meaningfully harmful. A continuous-variable approach would not be appropriate here because scores would include all rated ideas regardless of whether they are original, harmful, or both. This approach is inconsistent with the construct's definition requiring intentional harm in original ways (Harris et al., 2013). However, dichotomizing continuous variables results in information loss, reduces statistical power, and can attenuate observed relationships (MacCallum et al., 2002). Although this is common in creativity and MC research, future studies should explore alternatives. Another option is to use continuous self-report measures of malevolent behavior, although some of the most popular measures currently available have notable drawbacks, so there is still a need for a more appropriate measure (Cropley, 2026; Reiter-Palmon et al., in press).

Relatedly, the reliance on a single vignette in the primary analyses limits the generalizability of our findings. The data scientist scenario depicted interpersonal conflict primarily with a manager, which represents only one type of workplace provocation. As described in the Method, data from a second vignette depicting a nurse experiencing systemic organizational problems were excluded from the primary analyses due to distributional concerns with harmfulness ratings as a result of the vignette's inherent complexity.

The differences observed between the two vignette versions may reflect the difficulty of measuring MC in ecologically valid paradigms. Our ability to detect MC using threshold-based classification was constrained when the scenarios involved the potential for multidirectional harm or temporally contingent harm (e.g., short-term harm for long-term benefit). We believe this moral ambiguity was a result of the vignette's complexity. MC is defined by intentional harm, but intent was often not directly observable in participants' solutions. Following the CAT, raters are instructed not to infer meaning beyond what is explicitly stated, but evaluating intentional harm requires some inference about the problem solver's goal. The tension between the CAT (Amabile, 1982; Reiter-Palmon, 2021) methodology and the intentionality requirement of MC is a broader challenge for the field, as modified versions of the CAT are consistently used for MC scoring (e.g., Gutworth et al., 2018; Harris and Reiter-Palmon, 2015; Perchtold-Stefan et al., 2021; Nguyen et al., 2025). Although using explicit revenge tasks can help reduce this tension by predefining participants' harmful goals, raters must still evaluate the degree of harmful intent for each solution.

However, relying on explicit revenge tasks or prank scenarios where the harm may be easier to quantify does not solve the issue for measuring the role of problem construction in MC, where complex and ambiguous situations are required. Moreover, we argue that moral complexity is inherent in real-world problems, so ecologically valid tools should be designed to capture this complexity. Future research should develop measurement techniques capable of accommodating multidirectional harm. For example, raters can evaluate harm and benefit to specific stakeholders separately, or participants can report who they believe is harmed and benefited by their solution after generating ideas.

Another consideration for the generalizability of our findings is that we examined MC in retaliation to unfair and interpersonally provoking situations. Although injustice and interpersonal provocation are established situational antecedents of MC (e.g., Clark and James, 1999; Geng et al., 2024; Zhang et al., 2024), there may be other scenarios where individuals engage in MC. For example, individuals may engage in proactive MC for their personal gain, like the opening example of the Caltrain embezzlement case. In these cases, the perpetrators may not view their actions as harmful because they assist them in reaching their own goals. In other scenarios, harm may be recognized as harmful but ultimately viewed as collateral damage in pursuit of other neutral or benevolent goals.

In addition to motivation differences, different problem contexts, such as systemic organizational issues, peer conflicts, or customer-related frustrations, may elicit different patterns of problem construction and malevolent ideation. The relationship between problem construction fluency and MC observed here may not generalize to all workplace situations. Future research should examine whether these findings replicate across diverse workplace scenarios varying in complexity, as well as across contextual factors such as stakeholder involvement and the provocations experienced.

The present study is a first step in examining the cognitive underpinnings of the early stages of MC. However, our initial findings warrant a more in-depth investigation. The present study examined problem construction fluency, the number of unique problem restatements generated, as a predictor of MC. Although problem statement fluency is a well-established measure of problem construction engagement (Reiter-Palmon and Robinson, 2009), this approach treats all problem restatements equivalently, regardless of their content. Fluency is one measure that captures the output of problem construction engagement, not necessarily a direct operationalization of the level of cognitive restructuring. A richer analysis of problem construction could examine the valence of problem restatements themselves, the range of different themes of restatements (i.e., flexibility), or the quality or originality of problem restatements (Mumford et al., 1996). However, the same challenges we encountered in rating solution valence would likely apply to restatements: distinguishing harmful intent from neutral or constructive framing requires inference about goals that may not be explicit. Advancing MC research may therefore require the development of new methodological tools.

Although prior research has examined dispositional and situational predictors of malevolent creativity, few studies have directly assessed participants' intentions at the level of individual ideas, relying instead on inference from content alone. Therefore, methodological tools that researchers could develop include idea-level self-reports of intended harm. Options to avoid self-report bias could include forced-choice goal categorizations that require participants to specify who benefits and who is harmed by a given idea, or counterfactual assessments that distinguish intended harm from unintended or instrumental side effects. For example, participants can evaluate whether their ideas would still be acceptable or effective if they did not result in harm.

Another future direction is to examine the temporal dynamics of problem construction within the creative problem-solving process. Researchers could follow the problem statements or goals generated during problem construction and examine which ideas are further developed or integrated into solutions. Researchers could combine this approach with examining the order of restatements generated to help parse whether generating harmful ideas occurs more often early on or later in problem construction. It may be that individuals rule out harmful restatements early on as they explore more constructive options, or uncover them after failing to identify a clear goal path without causing harm.

In summary, the present findings highlight problem construction as a process associated with both benevolent and malevolent creative ideation. By showing that greater problem construction fluency is associated with harmful and malevolently creative ideas, this work provides preliminary empirical support for extending existing models of creative problem solving to MC. Clarifying how and when problem construction may be related to MC remains an important direction for future research.

## Data Availability

The datasets presented in this article are not readily available because participants did not consent to public access to their data. Requests to access the datasets should be directed to averielinnell@unomaha.edu.
