# Supplementary material for: Cognitive underpinnings of malevolent creativity: the role of problem construction
Source: Front Psychol. 2026 Sep 15;17:1788182. doi: 10.3389/fpsyg.2026.1788182 (PMC13619626; doi:10.3389/fpsyg.2026.1788182)
Supplement: Supplementary file 1 [file Supplementary_file_1.pdf]

Appendix A: Originality Scale

Instructions: Solutions should be rated on a 5-point scale based on the following criteria:

Novelty - Does the solution represent a relatively unique approach to the problem (relative to other solutions)?

Imagination - Does the solution present an imaginative or humorous approach?

Structure - Is the solution structured and limited by the problem as presented? Does the problem solver question the assumptions presented in the problem?

1. *Very unoriginal* - Simple solution with minimum effort.

2. *Unoriginal*- Simple but complete solution. One that is not novel, not imaginative, and is structured by the problem.

3. *Neutral* (neither unoriginal nor original) - Solution shows limited novelty or imagination, is still structured by the problem.

4. *Original* - Solution shows some novelty and imagination and is less structured by the problem.

5. *Very original* - Solution is unique and novel, imaginative, and not structured by the problem.

Appendix B: Valence Scale

Instructions: Solutions should be rated on a 5 point scale based on how **intentionally** benevolent or malevolent the idea would be to people, property, symbols, or processes.

Benevolence: Intention to do good or an act of kindness.

Malevolence: Intention to cause harm or evil.

1. *Very benevolent* – The solution intends to be very helpful or kind. The outcome would be a great amount of good.

2. *Moderately benevolent* – The solution intends to be somewhat helpful or kind. The outcome would be mostly good.

3. *Neutral* – The solution is neither intentionally harmful nor helpful. The outcome may result in some slight good or harm but would be overall neutral.

4. *Moderately malevolent* – The solution intends to be somewhat harmful. This harm could be recovered from.

5. *Very malevolent* – The solution intends to cause the most harm. This harm would be difficult to recover from.
